# Adult cognitive outcomes in phenylketonuria: explaining causes of variability beyond average Phe levels

**DOI:** 10.1186/s13023-019-1225-z

**Published:** 2019-11-28

**Authors:** Cristina Romani, Filippo Manti, Francesca Nardecchia, Federica Valentini, Nicoletta Fallarino, Claudia Carducci, Sabrina De Leo, Anita MacDonald, Liana Palermo, Vincenzo Leuzzi

**Affiliations:** 1 School of Life and Health Sciences, Aston University, Aston Triangle, Birmingham, England B4 7ET UK; 2 Department of Human Neuroscience – Unit of Child Neurology and Psychiatry, Sapienza University of Rome, Rome, Italy; 3 Department of Psychology, Sapienza University of Rome, Rome, Italy; 4 Department of Experimental Medicine, Sapienza University of Rome, Rome, Italy; 5 Department of Clinical Medicine, Policlinico Umberto I, Rome, Italy; 6 Birmingham Women’s and Children’s NHS Foundation Trust, Birmingham, UK; 7 Department of Medical and Surgical Sciences, Magna Graecia University of Catanzaro, Catanzaro, Italy

**Keywords:** PKU cognitive outcomes, Phe associations, Phe fluctuations, Phe variations, PKU guidelines

## Abstract

**Objective:**

The objective was to deepen the understanding of the causes of individual variability in phenylketonuria (PKU) by investigating which metabolic variables are most important for predicting cognitive outcomes (Phe average vs Phe variation) and by assessing the risk of cognitive impairment associated with adopting a more relaxed approach to the diet than is currently recommended.

**Method:**

We analysed associations between metabolic and cognitive measures in a mixed sample of English and Italian early-treated adults with PKU (*N* = 56). Metabolic measures were collected through childhood, adolescence and adulthood; cognitive measures were collected in adulthood. Metabolic measures included average Phe levels (average of median values for each year in a given period) and average Phe variations (average yearly standard deviations). Cognition was measured with IQ and a battery of cognitive tasks.

**Results:**

Phe variation was as important, if not more important, than Phe average in predicting adult outcomes and contributed independently. Phe variation was particularly detrimental in childhood. Together, childhood Phe variation and adult Phe average predicted around 40% of the variation in cognitive scores. Poor cognitive scores (> 1 SD from controls) occurred almost exclusively in individuals with poor metabolic control and the risk of poor scores was about 30% higher in individuals with Phe values exceeding recommended thresholds.

**Conclusions:**

Our results provide support for current European guidelines (average Phe value = < 360 μmol/l in childhood; = < 600 μmo/l from 12 years onwards), but they suggest an additional recommendation to maintain stable levels (possibly Phe SD = < 180 μmol/l throughout life).

**Public significance statements:**

We investigated the relationship between how well people with phenylketonuria control blood Phe throughout their life and their ability to carry out cognitive tasks in adulthood. We found that avoiding blood Phe peaks was as important if not more important that maintaining average low Phe levels. This was particularly essential in childhood. We also found that blood Phe levels above recommended European guidelines was associated with around 30% increase in the risk of poor cognitive outcomes.

## Background

Phenylketonuria (PKU; OMIM#261600) is an inherited metabolic disease where a genetic error results in a partial or complete de-activation of the enzyme phenylalanine hydroxylase (PAH) which normally metabolizes the amino acid phenylalanine (Phe; E.C. 1.14.16.1) into tyrosine (a precursor of dopamine). Phe accumulation results in several and still incompletely known negative effects on the postnatal development of the brain as well as on the functioning of the mature brain [1]. Fortunately, these negative consequences can be controlled by adopting, since birth, a Phe-restricted diet and protein supplementation. There is no question that a low Phe diet must be followed throughout childhood to achieve good cognitive outcomes [2]. However, several questions remain open [3]. We need to know more about: 1. which measures are most important to consider for dietary control (Phe average vs Phe fluctuations); 2. the impact of dietary control on different cognitive functions and possible interactions with age; and 3. which Phe value should be considered safe at different developmental age; there is uncertainty especially regarding the levels which are safe after early childhood. The purpose of this study is to provide some evidence relevant to these questions by analysing the performance of a mixed group of English and Italian early treated adults with PKU (from now on AwPKU) in relation to the current and historical blood phenylalanine control.

### Which metabolic measure? (average Phe levels vs Phe variation)

Blood Phe levels are usually measured with the assumption that they correlate with levels in the brain (see Leuzzi et al. [4]; Pietz et al. [5]; Rupp et al. [6], but also Brumm et al. [7], Moats et al. [8]; Schindeler et al. [9] for no relationship. Different measures of blood Phe have been found to correlate with cognitive performance, but their relative contribution is unclear (from now on Phe without qualification refers to blood Phe).

Most studies have assessed the impact of dietary control by considering either ***current Phe levels*** or ***average levels*** across a time period (also referred to as IDC- index of dietary control). Average levels have generally been calculated as a *mean* of *yearly median* values or, more rarely as a *mean* of *half-year median* values (for examples of this latter measure see Pietz et al. [10]; Vilaseca et al. [11]). These studies have shown that current Phe levels as well as average Phe levels are good predictors of cognition (for examples of positive associations in adults across cognitive functions see Brumm et al. [7]; Romani et al. [12]; for effects on IQ see Manti et al. [13]; Weglage et al. [14]; for effects on IQ in children, see Waisbren et al. [2]). Note, however, that effects are limited when only a restricted set of tasks is used [15, 16] and/or when only current Phe level has been considered; for example, effects of *current* Phe on IQ have been inconsistent across studies (see Jahja et al. [17]; Moyle et al. [18] for positive and/or marginal results; see Koch et al. [19]; Feldmann et al. [20]; Pietz et al. [10], for no correlation).

***Phe variation*** (also referred to as Phe fluctuation by some authors) has also been shown to predict cognition. Phe variation has generally been measured as a *mean* of *yearly SD* of Phe values [21–23]. Most studies have considered children and found that indexes of variation predict IQ (Burgard et al. [24]; Hood et al. [25]; marginally significant results in Anastasoaie et al. [21]; see also Vilaseca et al. [11] for results with a mixed-age group), executive functions [22, 24], motor control [26], white matter integrity [27]; for a review across functions, see Cleary et al. [28]. There is more limited evidence that Phe variation predicts cognitive outcomes long-term, since studies on adult patients are lacking.

Viau et al. [23] studied a mixed sample of children and young adults (*N* = 55) and assessed the impact of current and historical Phe on cognition. They reported limited correlations with Phe averages and no correlations at all with Phe SD. However, cognition was measured only with limited subtests from the WAIS and the WISC (Block design, Symbol Search and Verbal IQ or Verbal comprehension). Our previous study on a sample of 37 English AwPKU, early treated and with good metabolic control, showed significant effects of both historical Phe average and Phe SD (0–10, 11–16, 17+) on adult cognitive performance measured through IQ and an ad-hoc PKU battery of cognitive tasks [12].^1^ Importantly, however, these results did not provide information on the *relative* contribution of Phe average and Phe SD to cognitive outcomes. These two measures are, in principle, independent of one another. Two individuals can maintain the same average Phe level, but one may show little variation around the mean, with values very similar to one another, while another may show a lot of variation. Thus, both average Phe and Phe variation may contribute independently to good cognitive outcomes. However, in practice, these two measures are highly correlated in PKU populations, because individuals who maintain a lower Phe average, also maintain a more consistent low Phe diet [11, 12, 23, 25].

Hood et al. [25] reported some independent contributions of Phe SD, but they only assessed relationships in children and with limited cognitive measures (they found an independent contribution of childhood SD 5–10 at years or after 10 years on matrix reasoning and number of non-responses in an N-back task). In our study, we aim to assess an independent contribution of Phe SD on adult cognitive outcomes assessed more comprehensively.

### Individual variation in cognitive outcomes

While it is clear that cognitive outcomes depend on metabolic control, the extent of this dependence is debatable.

One question relates to whether all effects of having PKU can be eliminated through dietary control [1]. We know that most early-treated AwPKU perform within the norm, but that, as a group*,* their performance is worse than the controls. What we do not know, however, is whether the whole distribution of cognitive scores is shifted so that even performance at the high-end of the distribution is affected, or, instead, it is only the lower end of the distribution which is affected, where individuals are likely to have maintained poor dietary control. The first option will indicate that there are some fixed costs of having PKU which are not avoidable even maintaining a low Phe diet following current treatment guidelines. The second option instead, will indicate that a strict diet can completely eliminate the cognitive impact of having PKU.

A second, related question concerns the safe target range for blood Phe control at different ages. Current European guidelines advise to maintain Phe average levels below 360 μmol/L, before 12 years of age and below 600 μmol/L thereafter [29, 30]. American guidelines are even more strict recommending 120–360 μmol/L throughout life (American College of Medical Genetics and Genomics, ACMG) [31]. However, even the European guidelines have been criticized for being over stringent [32]. This is because there is little evidence of ill-effects when guidelines are relaxed in adulthood [13] and even the evidence to advocate childhood Phe < 360 is not strong [33–36]. A way to examine this question is to examine the distributions of cognitive scores within the PKU group in relation to metabolic control (see Waisbren et al. [2] for analyses of children data). This will allow us to examine if there are discontinuities in the distributions of cognitive scores, with pathological scores starting to appear and/or become more frequent when a given metabolic value is exceeded and whether these boundaries are consistent with current guidelines. Additionally, the cost of not following guidelines can be quantified by comparing the rates of poor cognitive scores in individuals which have or have not followed guidelines.

A final, related question, is whether there are individuals who have maintained poor metabolic control, but still have escaped cognitive impact. This will show that there is variability on how negatively PKU affects cognition (see van Vliet et al. [37] for a review of extreme case).

In conclusion, our study has two related aims: 1. To compare the effects of protracted exposure of brain to Phe –best measured through average Phe levels— with the effects of Phe peaks –best measures through SD from the mean--, and possible interactions with age. We want to see whether *both* average Phe and Phe SD contribute to adult outcomes and whether these two measures have a different weight in childhood and adolescence/adulthood. 2. To assess cognitive variability in a population of adults with PKU to see a) whether effects are pervasive or limited to a portion of individuals, b) whether the Phe boundaries identified by current European guideline are meaningful and c) whether there are exceptional cases where good cognition is achieved in spite of poor metabolic control.

To achieve aims, we have combined results from English and Italian AwPKU tested with the same battery of tasks (*N* = 56). Italian and English sub-samples show similar patterns of cognitive impairments and relationships with current and historical Phe measures, justifying accruing results (Romani et al., unpublished data). The resulting sample is larger and more varied in terms of metabolic control than most sets reported in the literature allowing better assessment of correlations between metabolic and cognitive variables (current Phe range is 54–2081; SD = 403; compared, for example, to: Brumm et al. [7]: 157–1713; SD = 338; Channon et al. [38]: 221–1233; SD = 261; Jahjia et al. [17]: 66–1550; SD = 342; Smith et al. [39]: 200–1879).

## Method

### Recruitment

Fifty six early-treated adult PKU participants were tested: 19 Italian and 37 English. They were all diagnosed soon after birth as result of national newborn screening programs.

The 19 Italian AwPKU were recruited from the Clinical Centre for Neurometabolic Diseases Department of Human Neuroscience, Child Neurology and Psychiatry Unit, Sapienza University of Rome. Three participants were currently treated with Kuvan. Nineteen Italian control participants were recruited among friends and students of the researchers. They were matched to the Italian PKU participants for age and education. Among the Italian participants, 4 had a diagnostic Phe level > 600 μmol/L but < 1200 μmol/L; 15 participants had Phe > 1200 μmol/L at birth.

The 37 English AwPKU participants were recruited from the Department of Inherited Metabolic Disorders at the University Hospitals Birmingham. They all had Phe > 1200 μmol/L at birth. The performance of this sample on a larger set of tasks as been described in previous publications [12, 40, 41]. Thirty English healthy controls were recruited through an advertising volunteering website. They were matched to the English PKU participants for age and education.

All AwPKU treated in the English and Italian centres were invited to participate and were accepted in the study on a first come, first served basis. The English study received NHS ethical approval. The Italian study was approved by the local ethics committee. All participants provided informed consent to the study.

### Metabolic measures

For both the English and the Italian PKU participants blood spots for blood Phe were taken regularly since diagnosis in early infancy and extensive records were available although there were limited data for a few participants (6 UK participants lacked or had very limited childhood data). We averaged Phe control in three age bands: childhood: 0–10 years old, adolescence: 11–16 years old, and adulthood: 17 years to present. We have also averaged measures throughout the life-time and considered current Phe level (for the Italian group, Phe has been measured immediately before the testing session/s or close to it; for the UK group, Phe has been measured immediately before the two testing sessions and averaged). We considered two types of measures: Phe average and Phe variation. Phe average in each band was calculated by taking the median values for each year and, then averaging the yearly values. The median is the value set halfway in a distribution of scores; it is generally used in the PKU literature rather than the mean because the median is not influenced by Phe variations. It is particularly, important to use the median in our study since we want to contrast a measure of central tendency (median, mean) with a measure of variation. Phe variation in each band was calculated by taking the SD for each year and then averaging yearly values in the band.

### Cognitive assessment

Cognitive assessments were carried out in a quiet room at the clinical centres in Birmingham and Rome by one the psychologist on the team**.** The testing session for the Italian participants lasted between 2 and 3 h. The English participants were tested in two separate sessions of similar length (a less extensive set of tasks was administered to the Italian participants because of resource limitations). A few PKU participants were not able to attend the second testing session which resulted in some data points missing for some tests (*N* = 31 instead of 37).

IQ was measured using, the Wechsler Adult Intelligence Scale-Revised (WAIS-R, [42]) with the Italian participants and the Wechsler abbreviated scale of intelligence (WASI, [43]) with the English participants, which includes the following subtests: Vocabulary, Block Design, Similarities, and Matrix Reasoning. In addition, participants were given a set of tasks chosen from the larger set of tasks administered in our previous studies [12, 40]. We chose tests which either showed a strong difference between participants with PKU and controls and/or strong correlations with metabolic measures. We also gave precedence to tasks with non-linguistic stimuli which did not need adapting across languages. Therefore, we did not include tests of picture naming, reading, spelling and orthographic knowledge (spoonerisms, phoneme deletions). Accuracy in these tasks was very good and not related to metabolic measures [12]. Speed of processing was assessed with visual search tasks. To reduce the number of tasks tapping similar functions, we also did not administer the Tower of Hanoi, the lexical learning task, the Stroop, and nonword repetition. Measures of STM (digit span and Corsi span) and a baseline measure of peripheral speed of processing were included for completeness and because of mixed results from the literature (for impairments in digit span and nonword repetition see Palermo et al. [40]; for contrasting results see Brumm et al. [7], and Moyle et al. [18]; see also Jahja et al. [17], for deficits with increasing working memory load).

The following cognitive areas were assessed:

1. ***Visual Attention.*** This was assessed with four tasks [12, 40]: 1.*Simple Detection*: Press a response button as soon as a ladybird appears on the screen; 2. *Detection with Distractors*: Press a button when a ladybird appears on the screen alone or with a green bug; in the second part of the task the instruction was changed to press a button when a green bug appears on the screen alone or with a ladybird; 3. *Feature Search*: Detect a target among distractors not sharing features by pressing a ʽyes’ or ʽno’ button (e.g., a red ladybird among green bugs)*; 4. Conjunction Search*: Detect a target among distractors sharing features (e.g., red ladybird among red bugs and green bugs). Both reaction times (RT from now on) and accuracy measures (error rates) were taken.
2. ***Visuo-motor Coordination***. This was assessed with two tasks: 1. *Grooved Pegboard Test* [44]: Put pegs into the holes of a board using only one hand as quickly as possible (short version with two trials one with the dominant and one with the non-dominant hand to match Italian and English samples) and 2. *Digit Symbol Task* [42]: Fill as many boxes as possible with symbols corresponding to numbers (key with associations remains visible) in 90 s. *Trail Making Test A (TMT A)* [45, 46]: connect circles containing numbers in ascending order of the numbers as quickly as possible.
3. ***Complex Executive Functions***. This was assessed with four tasks tapping skills such as planning, flexibility and abstract thinking: 1. *The Wisconsin Card Sorting Test (WCST)* 64 card version [47]*:* Discover the rules to match cards from a deck with four reference cards according to the shape, number or colour of the symbols on the card; feedback is provided to allow learning. Flexibility is required when the sorting rule is changed unknown to the participant and the new rule must be discovered. We used three different scores: total errors, number of perseverative responses and number of completed categories. 2. Difference in speed between *Trail Making Test B-A* (TMT B-A) [45, 46]. A involves connecting circles containing numbers in ascending order; B also involves connecting circles in ascending order, but alternating between circles containing numbers and letters . Only completion time is considered in this test; when, occasionally, an error is made, it is corrected by the examiner and this affects time to complete the task. 3 *Fluency:* For letter fluency: generate as many words as possible starting with a given letter in one minute of time (for Italian: P, F and L; Novelli et al. [48]; for English: C, F and L; Benton et al. [49]); for semantic fluency [50, 51]: generate as many names of animals as possible in one minute of time. This requires planning an efficient search through the lexicon.
4. ***Short-term Memory/Working Memory***. This was assessed with two tasks: 1. *Digit Span*: Repeat a sequence of digits spoken by the examiner, soon after presentation; 2. *Corsi Block Tapping Test* [52]: The examiner taps a sequence of blocks and the participant must reproduce the sequence in the same order.
5. ***Sustained Attention*** – This was assessed with the Rapid Visual Information Processing task (RVP; adapted from Sahakian et al. [53]): detect three target sequences of 3 digits by pressing the response key when the last number of the sequence appears on the screen. Scores are percentage correct.
6. ***Verbal Memory and Learning****.* This was assessed with *The Rey Auditory Verbal Learning Test* [54, 55] which asks for learning, immediate recall, and delayed recall of a list of 15 words. The list is presented five times and participants are asked to recall the words immediately after each presentation. After the 5th presentation (A5), an interfering list (B1) is presented and participants are asked to recall this list and then, once again, the original list (A6) without a further presentation. Finally, participants are asked to recall the original list after a 20-min filled interval. Our scores include total number of errors across the five learning trials (A1–5); errors in recalling the words after an interfering list (A6); and, again, errors in delayed recall of the original list.
7. ***Visual Memory and Learning****.* This was assessed with the *Paired Associates Visual Learning* [56]: Learn to associate objects with locations.

## Demographics and preliminary analyses

### Data analysis

For each participant, we computed z scores for each task using the relative (Italian or English) control group as reference. We also averaged z scores across tasks as a measure of overall cognitive performance. We report results of the PKU group using z-scores. Group differences of PKU from controls is examined through t-tests. Relationships between cognitive scores and Phe is examined with Pearson bivariate correlations. To reduce the number of variables per task, we did not carry out correlations with accuracy measures in search task (which are not impaired), and we only correlated for the TMT, the B-A condition; for the WCST, the total errors; and for the Rey, performance over 1–5 trials (learning) and in delay recall.

### Participants

Table 1 shows demographic variables for age, gender, years of education and Phe control across age. Average Phe level increased across ages (diet became more relaxed), Phe variation remained more stable (see also Hood et a [25]., for similar results in children up to 18 years old).

**Table 1:** Demographic and metabolic information for English and Italian PKU groups matched for age, gender and education, and for the whole group. Blood Phe measured in μmol/L

	PKU Group		Control Group	
	N = 56		*N* = 49	
	Mean	SD	Mean	SD
Age	**26.8**	*6.4*	**26.5**	*6.3*
Education (in years)	**14.3**	*1.9*	**14.7**	*1.8*
Gender (M/F)	**21//35**		**21//28**	
VIQ	**101.1**	*12.9*	**110.8**	*11.4*
PIQ	**102.7**	*15.2*	**110.1**	*11.1*
FIQ	**102.2**	*14.4*	**112.3**	*11.3*
Childhood (0–10 years)				
Phe Average Median	**457**	*213*		
Phe Fluctuation	**214**	*64*		
Mean N obs. Per participant	**201**	*141*		
Adolescence (11–16 years)				
Phe Average Median	**714**	*294*		
Phe Fluctuation	**162**	*56*		
Mean N obs. Per participant	**78**	*65*		
Adulthood (17 years +)				
Phe Average Median	**859**	*307*		
Phe Variation	**164**	*77*		
Mean N obs. Per participant	**63**	*67*		
Lifetime				
Phe Average Median	**655**	*263*		
Phe Fluctuation (SD)	**179**	*58*		
Mean N obs. Per participant	**341**	*213*		
Current Phe	**833**	*403*		
Range	65–2081			

### Cognitive outcomes

Cognitive performance across tasks is shown in Table 2. Patterns of results are very similar to those reported previously with an overlapping sample of 37 AwPKU [40], except for the visual paired-associate learning which shows a modest group impairment. The tasks with the largest differences from controls were tasks of visual search measured in terms of speed of processing and task involving visuo-motor coordination (pegboard, digit symbol, TMT A). Executive functions in terms of flexibility and planning, (TMT B, verbal fluency^2^) and sustained attention were also impaired consistent with previous results (see for *speed of processing:* Albrecht et al. [57]; *visuo-motor coordination:* Griffiths et al. [58]; Pietz et al. [10]; *executive functions:* Smith et al. [39]; Brumm et al. [7]; *sustained attention*: Schmidt at al [55].; Bik-Multanowski et al. [59]; Weglage et al. [14]; Jahja et al. [17]).

**Table 2:** Cognitive performance of the PKU group (English and Italian PKU participants; N = 56). Z scores calculated from respective control groups (*N* = 30 and *N* = 19). To facilitate interpretation, for all scores, higher Z-score reflect worse performance. Scores in bold are significantly higher than expected. ms. = milliseconds; sec. = seconds

	PKU Overall Z score		
	Mean	*SD*	Diff from controls (t-test)
IQ	**0.8**	*1.3*	***p*** **< .001**
Visual attention RTs			
Simple Detection - ms.	0.3	*1*	n.s.
Detention with Distractors – ms.	**0.8**	*1.6*	***p*** **< .01**
Feature Search – ms.	**1.5**	*2.3*	***p*** **< .001**
Conjunction Search m.	**1.2**	*1.7*	***p*** **< .001**
Visual attention accuracy			
Detention with Distractors - % errors	0.2	*1*	n.s.
Feature Search - % errors	0.2	*2.6*	n.s
Conjunction Search - % errors	−0.1	*0.8*	n.s
Visuo-motor coordination			
Pegboard (sec.)	**0.9**	*1.9*	***p*** **< .01**
Digit Symbol (%errors in 90 s.)	**0.7**	*1.2*	***p*** **< .01**
Trail-Making Test A: (sec.)	**0.8**	*1.9*	***p*** **< .01**
Executive functions			
WCST			
Total errors	0.4	*1.6*	n.s.
Perseverative responses	0.1	*1.1*	n.s.
N of Completed Categories	0.5	*1.9*	*p* = .06
Trail-Making Tests			
B (sec.)	**0.6**	*1.5*	***p*** **= .01**
B-A (sec.)	0.5	*1.7*	***p*** = .06
Verbal Fluency			
Letter (correct answers)	**0.6**	*1*	***p*** **< .01**
Semantic (correct answers)	**0.8**	*1.3*	***p*** **< .001**
Short term memory			
Digit span	0.3	*1.1*	n.s.
Corsi Block span	0.0	*1.1*	n.s.
Sustained attention			
RVP (% of errors)	**0.7**	*1.3*	***p*** **< .01**
Learning			
Rey Auditory Verbal Learning Test			
Trial A1-A5 (% errors)	0.2	*1.2*	n.s.
Retention (% errors A6)	0.3	*1.4*	n.s.
Delayed Recall (% errors)	0.3	*1.3*	n.s.
Paired Associate Visual learning			
(% errors)	**0.7**	*1.9*	***p*** **= .02**
Overall (excluding IQ)			
Mean	**0.5**	*0.8*	***p*** **< .001**

### Cognitive outcomes in relation to metabolic control

Table 3 shows bivariate Pearson r correlations between cognitive and metabolic measures. Correlations were extensive both for Phe average and Phe variations. Correlations were significant both with current and historical measures and for all tasks (except the Corsi span), although they were not systematic across all ages and types of metabolic measures. Significant correlations with lifetime measures (either average or SD) were found with IQ, speed in visual search, tasks tapping visuo-motor coordination, EF (WCST, TMT-B-A and semantic fluency), sustained attention, Rey words delayed recall, and paired visual learning.

**Table 3:** Pearson r correlations between Phe measures taken at different points in time and adult cognitive performance (N participants = 51–56; N tasks = 16). Significant correlations are in bold. ^a^ = significant <.05; ^b^ significant <.01. To facilitate interpretation, positive correlations always indicate that high Phe was associated with worse performance. Thus, for IQ, digit span, Corsi span, and semantic fluency correlations were reversed

PHE		Visual attention speed				Visuo Motor coordination		EF /Monitoring					Sustained	Learning and memory		
	FSIQ	Simple	Detection	Feature	Conjunction	Peg-	Digit	WCST	TMT	Digit	Corsi	Semantic	Attention	Rey	Rey	Paired Associate
		RT	with distractors RT	Search RT	Search RT	board	Symbol	Total errors	B-A	span	Span	Fluency	RVP %	a1-a5	delayed	Visual learning
0–10 yrs																
Average	.20	.22	**.30**^**a**^	**.33**^**a**^	**.34**^**a**^	.18	.18	.09	**.28**^**a**^	.12	−.02	.28	.12	.06	.07	**.34**^**a**^
SD	**.43**^**b**^	**.43**^**b**^	**.56**^**b**^	**.41**^**b**^	**.44**^**b**^	**.40**^**b**^	.15	.19	**.47**^**b**^	**.37**^**a**^	.06	.25	**.31**^**a**^	**.35**^**a**^	**.36**^**a**^	**.53**^**b**^
11–16 yrs																
Average	**.32**^**a**^	.14	.18	**.33**^**a**^	.27	**.28**^**a**^	.27	**.32**^**a**^	.22	.11	.09	**.34**^**a**^	**.32**^**a**^	.17	.14	**.60**^**b**^
SD	**.40**^**b**^	.16	.25	.25	.20	**.40**^**b**^	−.03	.21	.27	**.30**^**a**^	−.06	**.42**^**b**^	**.32**^**a**^	.07	.11	**.42**^**b**^
17 yr to now																
Average	**.42**^**b**^	.05	.**29**^**a**^	**.35**^**a**^	**.32**^**a**^	.24	**.35**^**a**^	**.31**^**a**^	**.43**^**b**^	.10	.05	.21	**.38**^**b**^	.28	**.29**^**a**^	**.44**^**b**^
SD	**.47**^**b**^	.06	.26	.23	.15	**.33**^**a**^	.00	.26	**.54**^**b**^	.12	−0.1	**.27**^**a**^	**.37**^**b**^	−.03	.06	.23
Lifetime																
Average	.23	.13	.27	**.40**^**b**^	**.40**^**b**^	.18	**0.28**	**.29**^**a**^	**.28**^**a**^	.01	.04	.16	.19	.12	.12	**.40**^**b**^
SD	**.56**^**b**^	.23	**.43**^**b**^	**.29**^**a**^	.24	**.39**^**b**^	0.07	**.28**^**a**^	**.47**^**b**^	.26	−0.04	**.29**^**a**^	**.40**^**b**^	.21	**.29**^**a**^	**.48**^**b**^
**Current**	**.43**^**b**^	.00	0.16	.09	.09	.26	**.34**^**a**^	.22	**.55**^**b**^	.05	−0.11	.17	**.42**^**b**^	**.34**^**a**^	**.39**^**b**^	**.33**^**a**^

Note. Correlations have been carried out using z scores. For the English PKU sample the number of participants and available Phe measures is slightly different across tests (range 31–37 participants), this means that the same value of r may have different probabilities

Consistent with previous results [12], tasks tapping visuo-attentional speed were associated with blood Phe early in life, but less with adult blood Phe and not at all with current Phe level. AwPKU who had maintained a more constant control in early childhood (0–10 years) still showed positive effects many years later, in adulthood, with faster RTs. In contrast, other tasks correlated strongly even with current Phe level. FSIQ, visuo-motor coordination (digit symbol), sustained attention, TMT B-A and learning are all strongly affected by current Phe level (as well as by levels at previous years).

## Phe average vs Phe SD

### Data analyses

Effects of Phe average and Phe SD were compared with different analyses. We compared the effect of these measures at different ages by contrasting correlations between Phe average/Phe SD in either childhood or adulthood and adult cognitive outcomes. We compared the number of significant correlations through χ^2^ tests and average size of correlation with t-tests.

Furthermore, we compared the relative contribution of Phe average and Phe SD to cognition by carrying out regression analyses where cognition was measured with either IQ or mean z-score in our cognitive battery as a summary measure of performance (contribution of individual measures is shown in the previous section with correlation analyses). We carried out three types of regressions. First of all, we compared the effects of Phe average and Phe variation across the lifespan. We carried out a two-steps regression where education was entered in the first step (to partial out any contribution) and both Phe average and Phe variation were entered together in the second step (forward method where the variables making the strongest contribution is considered first and, then, any other variable which makes an additional significant contribution is added). Note that entering education at a first step is a conservative choice, not only because there is a mutual relationship between IQ and education (with education influencing IQ, but also IQ influencing education), but also because Phe levels may influence education. In a second analysis, we assessed directly the contribution of Phe SD *after* Phe average was considered. Therefore, Phe average was forced in the first step and Phe variation was entered in the second step. Finally, we carried out a third type of regression to consider the contribution of metabolic measures at different ages. Based on the correlation results, we contrasted Phe average and Phe variation taken in childhood with the same measures taken either in adolescence or adulthood. All measures were entered together in the regression equation to see which combination predicted cognition best (SPSS forward method). In this analysis the order in which the variables are entered in the equation is identified by the regression model. The variable making a stronger contribution is entered first following by any other variable making an additional, significant contribution. We considered either adult or adolescent values in separate analyses because of their high correlation (for Phe average r = .74; for Phe variation r = .50) and becaue we wanted to avoid power with more variables.

### Results

#### Relative contribution of Phe average vs Phe SD in childhood vs adulthood

An inspection of Table 3 suggests that different metabolic measures have different effect on cognition at different ages. Considering ages further apart, we statistically compared correlations with Phe average and Phe SD in childhood and adulthood. Within childhood measures, there was a higher number of significant correlations and a higher mean correlation with Phe SD than with Phe average (12/16 vs 5/16; χ^2^ = 6.1; *p* = .01; Pearson r = .30 vs .16; t-test = 6.4; *p* < .001). The opposite was true for adult measures. Here, there was a higher number of significant correlations and a higher mean correlation with Phe average than with Phe SD (10/16 vs 5/16; χ^2^ = 3.1; *p* = .08; Pearson r = .27 vs .19; t-test = 2.3 *p* < .03). Importantly, the different degree of association of Phe variations with cognitive performance at different points in life was not due to differences in variability since Phe variation was similar across the life span (see Table 1). These results suggest that Phe average and Phe variation impact on the brain through different mechanisms since their effect differ at different ages. If this is the case, we should be able to demonstrate that both these measures contribute independently to explain adult cognitive outcomes.

#### Independent contribution of Phe average vs Phe SD

Results of regression analyses are shown in Tables 4 and 5. Table 4 shows two analyses. Analysis A is a two-steps regression where education was entered in the first step and Phe average and Phe SD were entered together in the second step. Results suggest that education influences IQ, but not cognitive performance in our battery (mean z-score). Crucially, results also show that Phe SD is the main predictor of both IQ and overall z-score. Phe average, however, also makes a (marginally significant) contribution to explain mean z-score. Analysis B is a two-steps regression where Phe average was entered in the first step and Phe SD in the second step. Results indicate that Phe SD is a significant predictor of performance even when the contribution of Phe average is partialled out, explaining a significant additional amount of variation both in IQ (27%) and mean z-score (23%). Phe average makes a smaller, marginally significant contribution, explaining 16% of variance in mean z-scores.

**Table 4:** Regression analyses predicting cognitive outcomes from Phe variation (SD) and Phe average entered either at the same step (forward method; set A) or at different steps (set b). Phe variation and Phe average are computed across the life-span. Coefficient and p refer to best model

Dependent variable		R2	Adj R2	Predictors	stand β	p	R2 change
Analysis-A							
Full IQ	Step 1	0.16	0.14	Education	.28	.01	
	Step 2	**0.39**	0.37	Phe SD	−0.5	<.001	
				Phe average	–	–	
Mean z-score	Step 1	0.09	0.08	Education	−19	.09	
	Step 2	**0.42**	0.39	Phe SD	.46	<.001	
				Phe average	.23	.049	
Analysis B							
Full IQ	Step 1	0.05	0.03	Phe average	−.03	n.s.	–
	Step 2	**0.32**	0.29	Phe SD	−.55	<.001	0.27
Mean z-score	Step 1	0.16	0.14	Phe average	.22	.06	–
	Step 2	**0.39**	0.36	Phe SD	.51	<.001	0.23

**Table 5:** Simultaneous regression analyses –method forward-- predicting cognitive outcomes from: a) Childhood Phe average; b) childhood Phe SD; c) adolescent/adult Phe average; d) adolescent/adult Phe SD. Analysis A uses adolescent measures; Analysis B uses adult measures

Dependent Variables	Predictors	Best model			
	Entered	R2	Adj R2	stand β	*p*
Analysis - A					
Full IQ	Adolescent Phe average	.23	.21	−0.48	.001
Mean z-score	Adolescent Phe average	.43	.41	0.38	.01
	Childhood Phe SD			0.36	.01
Analysis - B					
Full IQ	Adult Phe average	.22	.20	−0.47	.001
Mean z-score	Childhood Phe SD	.41	.39	0.41	.005
	Adult Phe average			0.32	.02

Table 5 shows the relative contribution of Phe measures (average and SD) taken either during childhood or adolescence/adulthood. Results show that IQ is predicted only by adolescent/adult Phe average. Mean z-score instead is predicted by *both* childhood Phe SD and adolescent/adult Phe average. These variables together predict a particularly high proportion of variance in mean z-score (41 and 43% respectively in a regression including either adolescent or adult Phe average).

## Conclusion

Overall these results highlight the importance of considering both Phe variation (especially in childhood) and Phe average levels (especially in adulthood) as predictors of adult cognitive outcomes.

## Individual variability in cognitive outcomes

### Data analyses

To examine individual variability in cognitive outcomes, we performed three different types of analyses.

First of all, we compared the distribution of cognitive scores in PKU and control participants to see if shifts in performance involved the whole distribution or only the bottom part of the distribution. For this purpose, we have divided both PKU and control groups into thirds according to best vs worse performance for IQ and mean z-score. Then, we have performed a between-subjects Anova with group (PKU vs control) and subgroup (top third vs bottom third) as independent variables and either IQ or mean z-score as a dependent variable.

Second, we examined the distribution of cognitive scores in relation to metabolic control. We first visually examined scatter plots to see if the Phe boundaries identified by current European guidelines were meaningful in eliminating/reducing individuals with poor scores. Then, we used χ^2^ tests to compare the proportions of individuals with poor cognitive scores whose metabolic control was or not within the guideline boundaries. Arbitrarily, we considered ‘poor’ scores, which were = > 1 z-score from the control group. In a normal distribution, this would apply to 16% of scores.

Lastly, we assessed if we could identify any exceptional cases in our data-base where poor Phe control was associated with good cognitive performance. To be conservative, we identified as having poor control any individuals with average Phe levels (in either childhood and adolescence) exceeding both European guidelines and the average for the PKU group (= > 500 in childhood and = > 900 in either adolescence and adulthood). To be conservative, we identified as having good cognition any individuals where IQ was within .05 z-score from controls, mean z-score was average (=0) or better than average (negative), and where there was a normal incidence of very poor z-scores (= > 1.5) across tasks (this proportion is on average 6% in the control group).

### Results

#### Distribution of cognitive scores in PKU and control groups

**Fig. 1:**
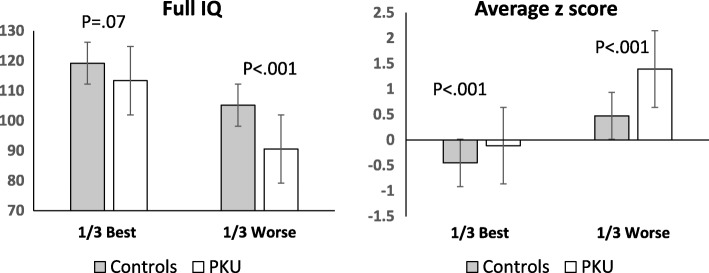
Differences between PKU and controls when individuals with best and worst performance in each group are compared (best 1/3 compared to worse 1/3). Bars show standard errors

Figure 1 compares results for the PKU and control subgroups depending on how their performance ranked within their group (best or worse third). Results were mixed. The PKU group was always worse than the control group, but differences were more marked for the worst subgroups. In fact, there was a significant interaction of group (PKU vs control) x subgroup (best vs worst third) with z-scores (F (1,65) =7.1; *p* = .01) and a marginal difference with IQ (F (1,65) =2.7; *p* = .10). These results indicate that, if there is a fixed cost of having PKU, this is marginal.

#### Distribution of scores in relation to metabolic control

**Fig. 2:**
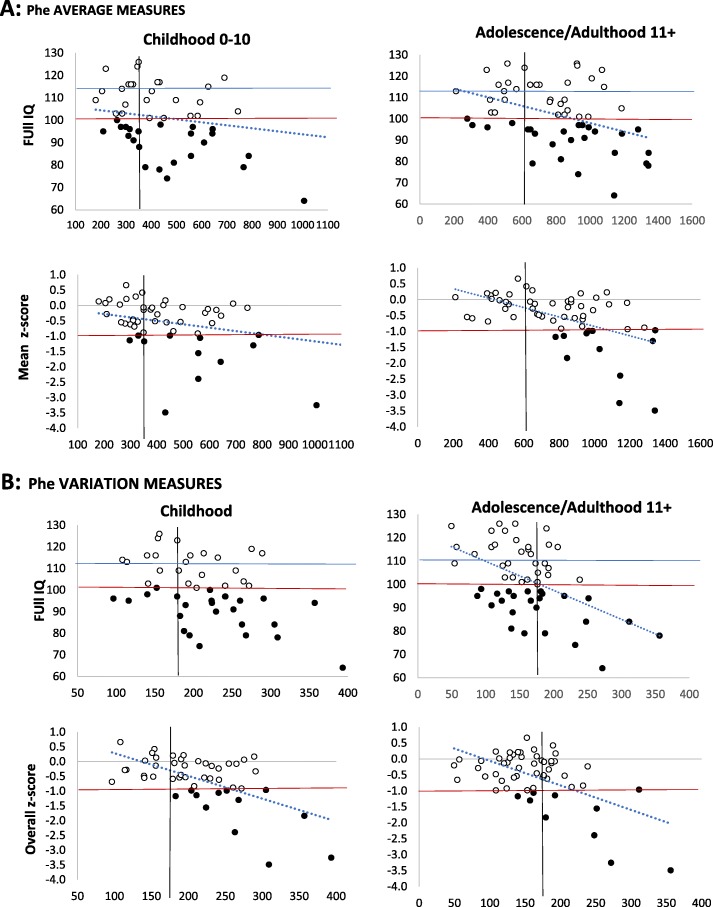
Cognitive outcomes in terms of IQ and overall z -score in our cognitive battery in relation to metabolic control. Panel A shows metabolic control in terms of Phe *average* levels in childhood and later on. Panel B shows metabolic control in term of Phe *variation* in childhood and later on. Note z-scores are depicted in figure so that both high IQ and high z-scores indicate good performance. The top horizontal line indicates average performance according to control group. The bottom horizontal line indicates performance = < 1SD from control average (FIQ for controls = 112.3; SD = 11.3; cut off 1 SD = 101). The black dots correspond to poor scores. The vertical lines indicate possible safety criteria (for Phe average < 360 in childhood < 600 afterwards; for SD < 180)

Figure 2 shows the distributions of IQ scores and z-scores in relation to metabolic measures: Phe average and Phe variation in childhood (Panel A) and Phe average and Phe variation in adolescence/adulthood (Panel B). To reduce the number of plots, we have averaged measures in adolescence and adulthood since these are highly correlated and similarly related to cognitive outcomes as shown by the regression analyses. With few exceptions, poor scores correspond to individuals who have not followed the guidelines (Phe levels = > 360 in childhood and/or = > 600 after that). Additionally, there are few or no individuals with poor z-scores and Phe variation < 180 in childhood and in adolescence/adulthood. There are more individuals with poor IQ and Phe variation < 180 in adolescence/adulthood, but note that even here Phe variation is a good predictor of performance as shown by the slope of the regression line.

Table 6 shows the number and % of individuals with poor cognitive scores in relation to guideline adherence. Numbers are small so exact values are not very meaningful, but patterns are clear. In all cases, there is a higher % of individuals with poor scores among those who have not followed guidelines and in most cases differences are significant. In particular, the risk of poor cognitive performance is about 30% higher in individuals who have relaxed the diet after childhood.^3^

**Table 6:** Number and percentages of poor cognitive scores in individuals who have followed or not followed European guidelines (Phe average < 360 in childhood; < 600 after that); Poor cognitive scores = > 1 SD worse than the controls. Z-score = mean z score in our cognitive battery

		Childhood 0–10				Adolescence/Adult 11+			
		Guidelines		Diff.	χ2	Guidelines		Diff.	χ2
		✓				✓			
Phe AVERAGE		Phe < 360	Phe > 360		p	Phe < 600	Phe > 600		p
IQ	N	10/22	16/28			4/15	23/41		
	%	**45.5**	**57.1**	**11.7**	.30	**26.7**	**56.1**	**29.4**	.05
z-score	N	3/22	9/28			0/15	12/41		
	%	**13.6**	**26.5**	**12.8**	.12	**0**	**29.3**	**29.3**	.01
Phe SD		Phe < 180	Phe > 180			Phe < 180	Phe > 180		
IQ	N	5/14	21/34			15/38	11/18		
	%	**35.7**	**61.8**	**26.1**	.09	**42.2**	**61.1**	**19.0**	.11
z-score	N	0/14	12/34			5/38	-7/18		
	%	**0**	**35.3**	**35.3**	.008	**13.6**	**38.9**	**25.7**	.03

#### Exceptional cases

In our sample, following our definition, there were 13 participants with poor metabolic control in childhood (average Phe= > 500); 12 with poor metabolic control in adolescence (average Phe= > 900) and 27 with poor metabolic control in adulthood (average Phe= > 900). Following our criteria, out of these participants, we could identify only 3 individuals with completely normal cognition and poor metabolic control in adulthood (3/27 = 11%). Their profile is shown in Table 7. We could not identify any individuals with poor control in childhood and adolescence and good cognition, but our samples are small. Also note that our definition of ‘good’ cognition was strict and included performance in our ad-hoc PKU cognitive battery. If we consider cognition only in terms of IQ, a larger proportion of participants have allegedly normal cognition (IQ within .5 SD from the control mean), but poor control in childhood: 4/13; adolescence: 4/12; and adulthood: 6/27. Across ages, we found 14/52 exception cases considering only IQ vs 3/54 considering cognition more widely (χ^2^ = 7.0; *p* = .008)**.**

**Table 7:** Performance of potentially exceptional participants with good cognition in spite of poor metabolic control or vice-versa poor cognition in spite of good control (see text for further explanation). Childhood Phe results for PKU108 are not available

	Good cognition but poor adult control		
	PKU4	PKU107	PKU108
	IT	UK	UK
Age	24	40	27
Education (in years)	13	14	16
Gender (M/F)	M	M	M
Full IQ	**109**	**117**	**126**
Feature search RT	−0.1	−0.3	−1.1
Conjoined search RT	0.1	−0.5	−1.4
Pegboard	0.1	−0.6	1.1
Average Z score	**0.1**	**0.0**	**−0.2**
% z score = > 1.5	**4.2**	**8.3**	**8.3**
Childhood (0–10 y)			
Phe Average Median	498	425	–
Phe Variation	181	213	–
Adolescence (11–16 y)			
Phe Average Median	634	622	845
Phe Variation	634	113	150
Adulthood (17 y +)			
Phe Average Median	**1067**	**1115**	**1000**
Phe Variation	239	114	137
Lifetime			
Phe Average Median	**672**	**978**	**975**
Phe Variation	169	148	139
Current Phe	1635	1080	685

## Discussion

Our study had two main aims: 1. to compare the effects of average Phe levels and Phe variation/fluctuation (in terms of Phe SD) on cognitive functions and 2. to explore cognitive variability in relation to metabolic control in a population of adults with PKU.

First of all, our results showed developmental interactions between type of cognitive function and type of metabolic measure (average vs SD). Different functions were affected by historical vs current metabolic control and by Phe average vs. Phe SD. Speed of processing in visual search was affected by metabolic control in childhood measured by both Phe median levels and Phe SD, while current Phe level had little impact [57]. Other tasks correlated significantly with current Phe --especially those involving visuo-motor coordination (digit symbol), learning and memory, an executive component (reasoning: IQ; flexibility: TMT B-A) and sustained attention (RVP). Consistent with our results, Moyle et al. [18] also found that a group of young adults with PKU (*N* = 12) showed significant differences from controls (N = 12) with the Processing Speed Index from the WAIS, but no correlations with *current* Phe level. The opposite was true for a memory index (WMS-III), where there was no impairment, but a strong correlation with *current* Phe levels (except for short-term memory, as in our case). Additionally, our regression analyses showed that, overall, adult cognition was best predicted by a combination of Phe childhood SD and Phe adolescence/adult averages.

These results suggest that there are different mechanisms through which Phe impact on cognition. High Phe may cause changes in levels of neurotransmitters (e.g., lowering dopamine) which can be modulated relatively short-term [60]. Alternatively, high Phe may affect brain structures (e.g., white matter; see Anderson et al. [61]; Anderson & Leuzzi, [62]) in a way which can be effective only during some critical periods and/or can be appreciated only long-term. Our results do not support one hypothesis of impairment versus another, but indicate that different mechanisms must be at play. This is necessary to explain our results showing that Phe average and Phe SD have a different impact on cognition at different ages. Possibly, Phe peaks are more detrimental for developing brains because they impact on white matter structural integrity [18, 63], while cumulative effects of Phe --as indexed by Phe averages—may be more detrimental for adult brains because they also impact on levels of neurotransmitters.

Clinically, our results indicate the importance of keeping low both Phe average and Phe variation. Regression analyses showed that Phe variation throughout the life-span was a stronger predictor of IQ and performance in our cognitive battery (mean z-score) although both Phe average and Phe variation across the life-span contributed to determine mean z-score. Together, childhood Phe variation and adult average levels accounted for as much as 41% of variability in overall z-score.

Regarding cognitive variability, our results confirmed extreme variability in cognitive outcomes across individual PKU participants. AwPKU in the top third of a distribution of cognitive scores performed very well. In fact, they performed almost as well as the control participants in the top third of their distribution. This suggests that the cognitive costs of PKU are minimal beyond those associated with high levels of Phe. If Phe levels are kept in check, performance can be (almost) as good as in the best controls. Minor costs could be associated to living with a chronic disease and/or with the fact that Phe control is not perfect even in individuals with top cognitive performance. This is in contrast with participants in the bottom third of the distribution who showed substantial impairments across a wide range of tasks.

Importantly, considering the distributions of cognitive scores in relation to metabolic control, we found that maintaining Phe < 600 after childhood significantly reduced the risk of poor cognitive outcomes by around 30%. This was true both when performance was measured by IQ and when it was measured as mean z-score in our cognitive battery. We also found that reduced Phe variation in childhood and afterwards significantly reduced the risk of poor outcomes (= > 1 z-score from controls) by a similar amount. These results, together with the results of our regression analyses, provide evidence for maintaining a good *and stable* dietary control after childhood. Distinguishing the contribution of metabolic control in adulthood and adolescence is more difficult given that these measures are highly inter-correlated. However, the significant effect of current Phe on specific tasks suggests that dietary control continuously modulates cognition, at least in some domains.

Our sample included a few individuals with poor metabolic control in adulthood, but excellent cognition. It is difficult to know if these individuals could have achieved even better cognition if they had maintained a low Phe diet, but these cases do raise the question of some possible protective factors in some individuals where the effects of high Phe levels are not evident. Reduced transport of Phe through the blood-brain-barrier could be one such factor although high levels of Phe were still found in the CSF of one these individuals described in the literature [64]. Recently van Vliet et al. [37] has reviewed exceptional cases from the literature of untreated individuals with PKU who have escaped mental disability. One has to stress, however, that these cases are very few. In our sample there were only three cases with good cognition and poor metabolic control in adulthood (Phe = > 900 μ/L; *N* = 3/27 = 11%) and no cases with good cognition and poor metabolic control at earlier ages.

Finally, we note the importance of using ad-hoc, comprehensive batteries to test and monitor cognition in people with PKU. Phe measures were more related to variance in our cognitive battery than in IQ (see Table 5). Moreover, the proportion of individuals who apparently achieved good cognition in spite of poor metabolic control was higher when cognition was measured only through IQ. It is important to consider cognition comprehensively to avoid false negatives of no consequences of relaxing the diet when, in fact, there is a clear impact in some cognitive areas.

### Study limitations

The main limitation of our study is the number of participants which interacts with the characteristics of metabolic control in samples of adults with PKU. It is difficult to evaluate whether metabolic control in adulthood contributes to cognitive outcomes above metabolic control in adolescence since these two measures are highly correlated. We need larger samples where these variables dissociate. Moreover, metabolic control is often good in childhood and adolescence and this reduces sample sizes when we want to assess the consequences of following the diet less strictly at these ages. Again, a larger sample will help to identify these cases. More collaborations across clinics are necessary to achieve adequate sample sizes.

### Conclusions

Our study has furthered our understanding of PKU in two main ways. We have demonstrated developmental effects where outcomes depend on complex interactions between the cognitive function examined, the age when metabolic control is measured (childhood, adolescence, current level) and the type of metabolic variable considered (Phe average vs Phe variation). Significant effect of childhood Phe levels on adult cognitive performance decades later support the idea of critical periods for brain development where high Phe levels and particularly Phe peaks (Phe variation) are very detrimental [65]. Significant correlations between *current* or *adult* average Phe levels and cognitive performance are consistent with the alternative, but not mutually exclusive, view that the toxic effect of Phe cumulate throughout the lifetime (for an overview on this topic see Berry et al. [66]).

Secondly, our study has provided evidence broadly consistent with the safety of following current European guidelines to avoid cognitive impairments. Levels < 360 μmo/l in childhood and < 600 μmo/l in adulthood prevent cognitive impairments. We have also suggested, however, that maintaining low Phe variation should be an equally important criterion when setting guidelines. In, our sample SD < 180 was also instrumental in preventing poor cognitive scores. While guidelines, should be followed to avoid poor outcomes, the presence of a few cases with high adult Phe level, but excellent cognition suggests that there is individual variability in Phe sensitivity. Given the social and economic costs of maintaining a low Phe diet, identifying who and why may avoid a strict diet without cognitive drawbacks should be one of the priorities of future research.

## Data Availability

The datasets used and/or analysed during the current study are available from the corresponding author on reasonable request.

## Appendix

**Table 8:** Raw scores for PKU (*N* = 50–56) and controls (N = 49); Highlighted results are significantly different between the two groups (see Table 2)

	PKU raw score		Controls raw score	
	Mean	*SD*	Mean	*SD*
Visual attention RTs				
Simple Detection - ms.	**329.3**	*49.9*	**315.5**	*52.0*
Detention with Distractors – ms.	**464.2**	*93.0*	**411.8**	*63.8*
Feature Search – ms.	**661.5**	*217.9*	**532.2**	*98.5*
Conjunction Search m.	**1062.7**	*246.6*	**878.3**	*165.4*
Visual attention accuracy				
Detention with Distractors - % errors	**0.8**	*1.0*	**0.6**	*0.9*
Feature Search - % errors	**1.6**	*3.5*	**1.5**	*2.1*
Conjunction Search - % errors	**2.2**	*3.5*	**1.6**	*4.5*
Visuo-motor coordination				
Pegboard (sec.)	**73.6**	*13.1*	**67.6**	*7.9*
Digit Symbol (%errors in 90 s.)	**38.9**	*11.7*	**26.0**	*10.0*
Trail-Making Test A: (sec.)	**27.6**	*12.0*	**23.8**	*9.6*
Executive functions				
WCST				
Total errors	**13.9**	*7.7*	**11.9**	*5.2*
Perseverative responses	**7.7**	*4.5*	**7.2**	*4.0*
N of Completed Categories	**3.9**	*1.1*	**4.3**	*0.9*
Trail-Making Tests				
B (sec.)	**56.2**	*29.2*	**45.9**	*17.5*
B-A (sec.)	**28.7**	*23.2*	**22.1**	*13.2*
Verbal Fluency				
Letter (correct answers)	**37.0**	*11.1*	**44.0**	*11.9*
Semantic (correct answers)	**20.9**	*5.7*	**24.4**	*4.52*
Short term memory				
Digit span	**6.0**	*1.0*	**6.3**	*1.0*
Corsi Block span	**5.5**	*0.9*	**5.4**	*1.0*
Sustained attention				
RVP (% of errors)	**20.6**	11.8	**14.3**	*9.1*
Learning				
Rey Auditory Verbal Learning Test				
Trial A1-A5 (% errors)	**22.8**	*10.4*	**20.2**	*7.5*
Retention (% errors A6)	**17.9**	*14.5*	**12.9**	*13.8*
Delayed Recall (% errors)	**14.0**	*14.3*	**11.2**	*13.4*
Paired Associate Visual learning				
(% errors)	**2.8**	3.3	**1.7**	*1.7*

## Abbreviations

AwPKU: Adults with PKU
EF: Executive functions
Phe: Phenylalanine
PKU: Phenylketonuria
RVP: Rapid visual processing
SD: Standard Deviation
STM: Short-term memory
TMT: Trial making test
WCST: Wisconsin card sorting test
μmol/L: Micromole per liter

## Footnotes

1 Life-time Phe SD predicted FIQ and visuo-motor coordination; childhood Phe SD predicted visuo-spatial attention, complex EF and memory and learning; adolescence Phe SD predicted spoken language and adult Phe SD predicted sustained attention.

2 Note these tasks have also a speed component, although not as strong as tasks measuring RTs.

3 Results were in the right direction but did not reach significance when we considered childhood average levels. This may simply be error variation due to our small sample; childhood Phe average, in fact, was also significantly correlated with adult outcomes.
